# Using structural equation modeling to examine the influence of family planning social norms on modern contraceptive use in Nigeria

**DOI:** 10.3389/fsoc.2022.866254

**Published:** 2022-10-20

**Authors:** Mahua Mandal, Lisa M. Calhoun, Courtney McGuire, Ilene S. Speizer

**Affiliations:** ^1^Carolina Population Center, University of North Carolina at Chapel Hill, Chapel Hill, NC, United States; ^2^Department of Maternal and Child Health, Gillings School of Global Public Health, University of North Carolina at Chapel Hill, Chapel Hill, NC, United States

**Keywords:** family planning, social norms, Nigeria, Nigerian Urban Reproductive Health Initiative, structural equation modeling, gender norms

## Abstract

Despite high knowledge of family planning (FP) among Nigerian women, use of modern contraceptives remains low. While FP investments in Nigeria have been ongoing for decades, relatively little emphasis on contextual and structural factors may have contributed to low demand for and use of contraception. From 2009 to 2014, the Bill & Melinda Gates Foundation (BMGF) supported the Nigerian Urban Reproductive Health Initiative (NURHI) with the aim of increasing voluntary use of contraceptives among women ages 15–49 years in six Nigerian cities. A subsequent phase of NURHI was implemented in three states for the next 3 to 5 years. Using cross-sectional survey data from three cities (two exposed to NURHI, one not exposed), this study examines whether social norms around FP were related to women's use of modern contraception, and whether the relationship differed by varying levels of exposure to the program (i.e., by city). We identified three distinct FP social norms through factor analysis: norms around delaying first pregnancy; spacing or limiting pregnancies; and using contraception when the husband disagrees. Using structural equation modeling, we found that FP social norms are related to use of modern contraceptive methods, and the relationship varies by city and norm type. The observed differences suggest that this relationship depends on numerous factors at the individual, interpersonal and societal level, and this may include malleable factors influenced by the NURHI program.

## Introduction

Nigeria's population, estimated at about 200 million as of 2019, is projected to increase to about 440 million by the year 2050 (United Nations, 2019). With a current rate of over 5 children per woman, Nigeria's total fertility rate remains higher than most other sub-Saharan African countries, including those in West Africa (United Nations, 2019). Fertility levels are particularly high in some Nigerian subpopulations, including among residents of the North West and North East regions of the country; Hausa, Fulani, and Kanuri ethnic groups; and those who identify as Muslim or practice traditional religions (Mberu and Reed, 2014). Despite high knowledge of family planning (FP) in Nigeria-−93% of women of reproductive age know of at least one contraceptive method—contraceptive use remains low (National Population Commission (NPC) [Nigeria] and ICF International, 2019). Among married Nigerian women modern contraceptive use increased from 10% in 2013 to only 12% in 2018 (National Population Commission (NPC) [Nigeria] and ICF International, 2014, 2019). There is wide geographical variation in modern contraceptive use in Nigeria, with estimates among married women ranging from 3.9% in the North-West region to 20.0% and 25.4% in the North-Central and South-West regions, respectively (National Population Commission (NPC) [Nigeria] and ICF International, 2014).

A plethora of studies have examined individual- and inter-personal level predictors of contraceptive use in Nigeria. These include wealth, education, ethnicity, women's decision-making abilities, and spousal support and communication (Achana et al., 2015; Ezeanolue et al., 2015; Adebowale et al., 2016; Wulifan et al., 2016; Asaolu et al., 2017; Alo et al., 2020; Bolarinwa et al., 2021). A smaller body of research has examined health facility- and community-level factors, including method stock out (Anglewicz et al., 2021), health care worker home visits (Asaolu et al., 2019), social norms (Alo et al., 2020), and community-level knowledge and literacy (Bolarinwa et al., 2021), that influence use of contraception. While FP investments in Nigeria have been ongoing for decades, relatively little emphasis on contextual and structural factors may have contributed to low demand for and use of family planning (Ejembi et al., 2015). Greater focus on structural influences, including social norms, that increase acceptance and use of contraceptive methods is needed.

Social norms are informal rules that govern behavior in a particular context (Cialdini et al., 1991). Scholars from across the social sciences have attempted to measure and explain the effect of social norms on people's choices and behaviors (Cialdini et al., 1991; Boyd and Richerson, 1994; Brennan et al., 2013; Elsenbroich and Gilbert, 2014; Young, 2015). While collective (social) norms exist at the social level—typically, the group, community, or national level—individuals' interpretation of these norms, known as perceived (social) norms, exist at the individual, psychological level. Perceived norms are the result of individuals' cognitive processes; thus, understanding the role that social and behavior change communication programs have on influencing norms is operationalized through measurement of perceived norms (Lapinksi, 2005). While some health-related studies have attempted to measure the influence of collective norms on health choices and behaviors (Babalola, 2007; Rimal et al., 2013; Sedlander and Rimal, 2019), research on family planning and sexual and reproductive health has increasingly focused on the ways in which perceived norms influence behaviors (Dynes et al., 2012; Rimal et al., 2015; Jain et al., 2018; Cislaghi and Heise, 2019b; Costenbader et al., 2019).

Perceived norms are further delineated into injunctive norms—beliefs about what other people approve of or think one should do—and descriptive norms—beliefs about what other people do (Cialdini et al., 1991; Cialdini and Trost, 1998). The influence of injunctive and descriptive norms on family planning and reproductive health behaviors is mixed. For example, one study evaluating the influence of a male engagement and social norms intervention in the Democratic Republic of Congo found that injunctive norms among married women and descriptive FP norms among married men were associated with future intention to use FP. However, there was no association between descriptive FP norms among women, or injunctive FP norms among men, and future intentions to use FP (Costenbader et al., 2019). Another study in Ethiopia found that, among 15–24 year old male youth, the descriptive norm of knowing a friend who had ever used condoms was associated with use of condoms at last sex, and the injunctive norm of being worried about other people would think if the respondent needed condoms significantly decreased their likelihood of using condoms (Jain et al., 2018). Another study implemented in Kenya and Ethiopia found that injunctive norms alone were not associated with women's contraceptive use; rather, women whose current number of sons was lower than their perception of the community ideal had a lower odds of reporting contraceptive use, while women whose personal ideal number of sons was lower than their perceived community ideals had a greater odds of reported contraceptive use (Dynes et al., 2012).

The Nigerian Urban Reproductive Health Initiative (NURHI), funded by the Bill and Melinda Gates Foundation (BMGF) and implemented by the Johns Hopkins Center for Communication Programs, aimed to increase voluntary modern contraceptives use among women ages 15–49 years through comprehensive demand and supply side programming. NURHI programming was guided by the theory of ideation, which proposes that people's actions are influenced strongly by their beliefs, ideas, and feelings. Ideation factors include personal attitudes and beliefs (i.e., what a person knows about FP and how they think it will affect them), and social norms (i.e., what a person believes other people will think of them if they use FP). NUHRI designed and integrated communication methodologies, including those used in mass media campaigns and social mobilization efforts, into each component of the program, including the service delivery ones; and placed intensive and sustained effort and resources into demand generation activities. NUHRI defined demand for family planning as the desire and ability among women and/or men to take action to plan their facilities. The program hypothesized that the demand generation elements would work together to influence ideation factors, including social norms; and these, along with the supply programming, would in turn increase use of modern contraception (Krenn et al., 2014).

Phase 1 of NURHI was implemented from 2009 to 2014 in six Nigerian cities: Abuja, Ibadan, Ilorin, Kaduna, Benin and Zaira. NURHI's demand generation activities consisted of communication campaigns to promote discussion of FP, reduce social barriers, myths, and social stigma, and increase approval of FP methods. Vehicles for the communication campaign included mass media (posters, television and radio spots), entertainment-education (radio and television dramas), and social mobilization to enhance interpersonal communication during client-provider interactions, between spouses, during trade group meetings, and through neighborhood campaigns and social events (NURHI, no datea). Supply side programming included provider training, ensuring security of commodity supplies, and improving the overall clinic environment in target facilities (NURHI, no dateb). A subsequent phase of NURHI, Phase 2, was implemented in three states: Oyo, from 2015 to 2018; and Lagos and Kaduna from 2015 to 2020. Phase 2 focused on specific priority audiences of women with unmet need, traditional method users, men and service providers (NURHI, 2018a); and integrated the use of digital and social media into the demand generation strategy (NURHI 2, no date). For further details on the NURHI intervention, please see Krenn et al. (2014) and Adedini et al. (2018).

This study examines whether social norms around FP are related to women's use of modern contraception. Focusing on residents in the cities of Ilorin, Kaduna and Jos, this study also examines whether the relationship between social norms and use of modern contraception differs by varying levels of exposure to NURHI (i.e., by city).

## Materials and methods

### Study design and sample

The NURHI Sustainability Study examined the continued impacts of the NURHI program on FP attitudes and behaviors 2 years after the end of Phase 1. We used data from the 2017 cross-sectional survey that was part of a 2015 parent study. Three cities with varying levels of the program were included in the current analysis. NURHI Phase 1 only was implemented in Ilorin; NURHI Phases 1 and 2 were implemented in Kaduna; and in Jos, no NURHI program had been implemented.

In 2015, a cross sectional survey was undertaken in Ilorin and Kaduna. For the 2015 survey, a two-stage sampling design was used to obtain a representative sample of respondents in each city. First, enumeration areas from the 2006 Nigeria census frame were grouped into primary sampling units (PSUs); a random selection of PSUs was then taken. Next, a household listing and mapping was undertaken and then 41 households were randomly selected from each PSU. Following informed consent, all women of reproductive age (15–49 years) who had spent the previous night in the household were eligible for participation. The 2015 data were not utilized for the current analysis, but all of the PSUs in Ilorin and Kaduna in the 2015 survey were included in the 2017 survey.

In 2017, a second cross-sectional survey was undertaken which included Ilorin, Kaduna and Jos. In Ilorin and Kaduna, we undertook a census of all households located in the sampled PSUs from the 2015 survey to permit matching women to the 2015 sample. All women ages 15–49 who had spent the previous night in the household were eligible to participate in the study after providing informed consent. In Jos, which was not included in the 2015 survey, a two-stage sampling design was used. A total of 56 PSUs were selected from the 2006 Nigeria Census sampling frame. A listing and mapping exercise was undertaken and a random sample of 33 households was selected in each Jos cluster. All women ages 15–49 years residing in or visiting the selected households the night before the survey were eligible to be interviewed after providing informed consent. The initial sample size was 10,535. The analysis in this paper includes only women who reported ever having sexual intercourse. After dropping respondents who reported never having sexual intercourse, the final sample size for this study was 6,396 (1,685 in Ilorin; 3,238 in Kaduna; and 1,473 in Jos).

### Study measures

#### Outcome measure: Use of a modern contraceptive method

The 2017 NURHI Sustainability Study survey included a question on which contraceptive methods the respondent or her partner was using at the time of the survey. Modern method use consists of use of at least one the following: female sterilization, implant, intrauterine device (IUD), injectables, daily pill, emergency pill, male condom, female condom, lactational amenorrhea method (LAM), or standard days method (SDM). Women were coded one if they currently used any of these modern methods and zero otherwise.

#### Social norms

The survey included a series of quantitative questions based on vignettes to elicit social norms related to FP use for delaying first pregnancy, spacing pregnancies, and limiting pregnancy. Vignettes are mini-scenarios that ask respondents about their perspectives and attitudes toward one or more fictional characters, and are increasingly used to measure social norms (Cislaghi and Heise, 2016; Learning Collaborative to Advance Normative Change, 2019). The study vignettes included (1) an adolescent girl who was sexually active with her 17-year-old boyfriend and was considering using modern contraception; (2) a 21-year-old mother of a 6-month-old baby who wanted to space her next pregnancy while her husband wanted another child immediately; and (3) a 28-year-old woman with four children who wanted to prevent future pregnancies. All social norms survey items were asked on a five-point Likert response scale (e.g., strongly agree, agree, do not agree nor disagree, disagree, strongly disagree) (see social norms survey items on Tables 2, 3).

#### Individual-level factors

Respondent characteristics included age (categorical, in 5-year increments), marital status (ever married/living together, never married/living together), highest education level (none, Quranic only, primary, junior secondary, senior secondary, higher), parity (none, 1–3, 4–6), religion (Christian, Muslim), religiosity (not at all or somewhat religious, strongly religious), and wealth based on quintile levels. These factors were included given the extant evidence on their relationship to use of modern contraception (Achana et al., 2015; Ezeanolue et al., 2015; Adebowale et al., 2016; Wulifan et al., 2016; Asaolu et al., 2017; Alo et al., 2020; Bolarinwa et al., 2021).

### Data analysis

All analyses used weighted data and accounted for the clustered design of the 2017 NURHI Sustainability Study. First, we explored the demographic characteristics of the sample, calculating frequency distributions for the whole sample and by location. Next, we conducted exploratory factor analysis (EFA) with oblique (Promax) rotations to determine the latent constructs of social norms. We used scree tests and eigenvalues to determine the number of social norm factors to retain. We dropped items that produced factor loadings below 0.45 or uniquenesses (i.e., percent variance unexplained) above 0.75, and that did not conceptually fit the factor model. To examine the strength of each FP social norm, we calculated mean scores for each factor by multiplying the items within the factor by their factor loading. We then summed across the products and divided by the number of items within the factor. Last, we fit generalized structural equation models to assess whether FP social norms were associated with respondents' use of modern contraceptives, and whether the relationship differed by city. We fit several models using data from the full sample and used Akaike Information Criterion (AIC) to determine whether one model fit the data better than others. Subsequently, we used a series of tests of invariance to conduct group comparisons of the selected model by city.

### Ethics

The study protocol and all consent procedures and consent forms were approved by the Institutional Review Board at the University of North Carolina at Chapel Hill and by the National Health Research Ethics Committee of Nigeria (NHREC) in Nigeria.

## Results

Across all cities, sexually experienced respondents were, on average, 32 years old, and about 84% had been ever married or lived with a man as married. Senior secondary school was the highest level of education for about one-third of women. About 20% of women had no children, almost half (44%) had between one child and three children, and the remaining had four to six children. The sample of women who ever had sex from Jos is slightly younger, less likely to be ever married or living with their partner, and have fewer children than their counterparts from Kaduna or Ilorin. In Ilorin and Kaduna almost two-thirds of respondents were Muslim (66% and 57%, respectively), while in Jos almost two-thirds were Christian (64%). More than three-quarters of respondents reported being strongly religious (81% in total). About 30% of women had used a modern method of contraception at the time of the survey (see Table 1).

**Table 1:** Demographic characteristics and use of modern contraceptive methods among 15–49 year old female respondents in Nigeria who ever had sex, 2017 (*n* = 6,396).

	**Ilorin**		**Kaduna**		**Jos**		**Total**	
	**(*****n*** = **1,685)**		**(*****n*** = **3,238)**		**(*****n*** = **1,473)**		**(*****n*** = **6,396)**	
**Age**								
Mean (Median)	32.3 (32.0)		32.1 (31.0)		31.3 (30.0)		32.0 (31.0)	
	**Percent** ^*^	**n** ^¥^	**Percent** ^*^	**n** ^¥^	**Percent** ^*^	**n** ^¥^	**Percent** ^*^	**n** ^¥^
**Age in 5-yrs increments**								
15–19 years	4.9	85	3.8	119	5.2	79	4.5	283
20–24 years 25–29 years 30–34 years 35–39 years 40–44 years 45–49 years	14.3 21.3 17.6 19.2 12.6 10.1	241 355 293 324 215 172	15.9 22.4 19.6 16.4 12.5 9.5	523 732 619 539 397 309	16.7 23.3 20.0 16.7 9.8 8.3	241 352 292 249 139 121	15.5 22.2 19.0 17.6 11.9 9.4	1,005 1,439 1,204 1,112 751 602
**Marital status**								
Ever married/living together Never married/living together	84.2 15.8	1,417 268	86.7 13.3	2,850 388	78.9 21.1	1,166 307	83.9 16.1	5,433 963
**Highest level of education**								
None Quranic only Primary Junior secondary Senior secondary Higher	7.4 2.2 16.6 3.2 35.5 35.2	126 35 282 58 595 588	3.0 8.9 14.9 11.2 33.5 28.5	86 314 495 386 1,074 883	3.6 5.3 13.7 13.0 31.4 32.9	46 76 202 191 474 483	4.8 5.5 15.2 8.7 33.7 32.0	258 425 979 635 2,143 1,954
**Parity**								
0 1–3 4–6	18.5 44.2 37.2	313 740 632	17.8 43.2 39.1	544 1,382 1,312	23.9 44.0 32.2	348 633 492	19.5 43.7 36.9	1,205 2,755 2,436
**Religion**								
Christian Muslim	34.2 65.8	582 1,103	42.9 57.1	1,144 2,094	64.3 35.7	943 50	44.7 55.3	2,669 3,727
**Religiosity**								
Not at all or somewhat Strongly	20.9 79.1	356 1,329	17.2 82.8	529 2,709	19.3 80.7	282 1,191	19.1 80.9	1,167 5,229
**Wealth**								
Poorest Poor Middle Wealthy Wealthiest	19.6 21.3 21.4 20.8 16.9	331 357 358 351 288	17.1 19.4 19.9 20.8 22.8	542 633 664 691 708	17.3 21.3 20.1 20.1 21.2	258 290 290 319 316	18.1 20.6 20.5 20.7 20.2	1,131 1,280 1,312 1,261 1,312
Use of modern method of contraception	31.3	529	30.7	946	28.7	427	30.4	1,909

Percentages are adjusted for the design of the survey.

* Weighted percent.

¥ Unweighted n.

Results from weighted exploratory factor analysis revealed three social norm factors, or constructs, around FP. The first factor included three items about FP social norms to delay first pregnancy among adolescents (alpha = 0.68). The second included four items about FP social norms to space pregnancies or limit all pregnancies among married women (alpha = 0.79). The third included three items about FP social norms around women's use of contraception when their husbands disagree (alpha = 0.75). Three survey items about FP social norms that did not conceptually or statistically fit were discarded from the final factor model (see Tables 2, 3).

**Table 2:** Factor loadings and uniqueness's based on exploratory factor analysis for items related to social norms around family planning in Nigeria, 2017 (*n* = 6,396).

	**Expectations and community approval of adolescents' contraceptive use to delay first pregnancy**	**Community approval for spacing and limiting births**	**Expectations and community approval of contraceptive use when husband does not agree**	**Uniqueness**
	**(α = 0.68)**	**(α = 0.79)**	**(α = 0.75)**	
**Factor loadings**	
Belief that most community members would say a sexually active unmarried adolescent should use modern contraception to avoid pregnancy [sn2]	0.70			0.54
Belief that most sexually active unmarried adolescents in community would use contraception [sn3]	0.71			0.51
Belief that most community members would say that a married adolescent who does not want to get pregnant should use modern contraception [sn4]	0.47			0.71
Belief that most community members would approve of a young woman with an infant spacing her next pregnancy [sn5]		0.61		0.58
Belief that most community members would agree that a young woman with an infant who wants to space her next pregnancy should use modern contraception [sn6]		0.60		0.55
Belief that most community members would approve of a woman with four children who wants no more to prevent another pregnancy [sn10]		0.76		0.48
Belief that most community members would agree that a woman with four children who wants to prevent another pregnancy should use contraception [sn11]		0.74		0.47
Belief that most community members would agree that a woman with an infant who wants to space her next pregnancy should use contraception even if her husband does not want her to [sn7]			0.80	0.41
Belief that most women with infants who want to space their next pregnancy would use contraception even if their husbands did not want them to [sn8]			0.68	0.48
Belief that most members of a woman's religious congregation would approve of her contraceptive use even if her husband wanted another baby soon [sn9]			0.59	0.66

**Table 3:** FP social norm survey items discarded from the final three-factor model.

Belief that community members would say an unmarried adolescent with a boyfriend should not have sex [sn1]
Belief that most community members would think a woman has concerns about having more children should talk to her husband [sn12]
Belief that a health care provider who learns that a woman is seeking contraception without her husband's knowledge would give the woman contraception [sn13]

Mean scores for each factor showed that, overall, FP social norms around spacing and limiting pregnancies was strongest, or most positive, while norms around delaying first pregnancy was weakest, or least positive. Mean scores differed slightly by city, with FP social norms for delaying first pregnancy highest in Kaduna and lowest in Jos; for spacing and limiting pregnancies highest in Ilorin and lowest in Jos; and for using contraception when the husband disagreed highest in Ilorin and lowest in Kaduna (see Table 4).

**Table 4:** Mean score of FP social norm factors by city.

**Mean score of FP social norms**	**Ilorin** ** (*n* = 1,685)**	**Kaduna** ** (*n* = 3,238)**	**Jos** ** (*n* = 1,473)**
Delaying first pregnancy	1.69	1.72	1.67
Spacing and limiting pregnancies	2.91	2.74	2.58
Using contraception when husband disagrees	2.03	1.97	2.01

**Figure 1:**
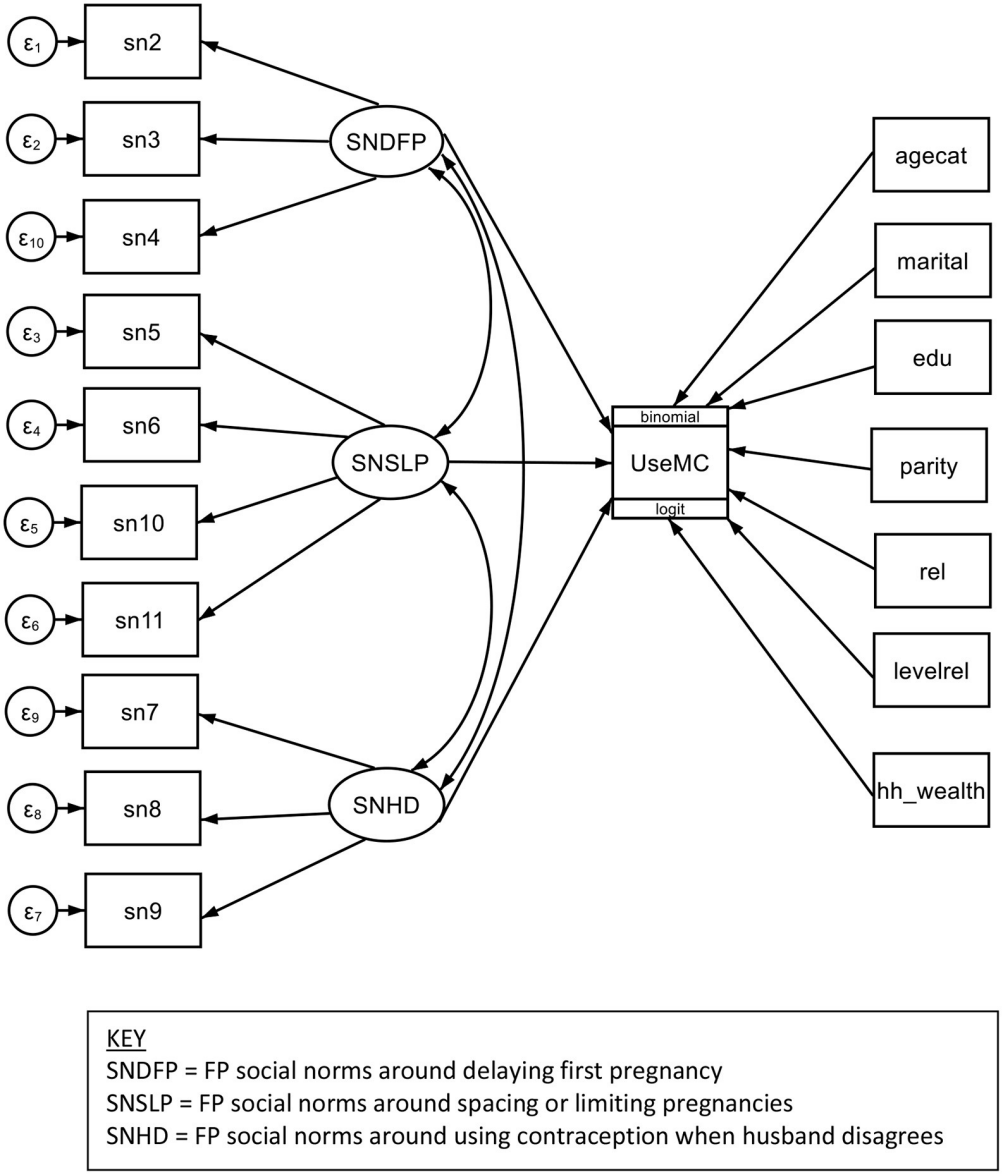
Structural equation model for association of family planning social norms with current use of modern contraceptive methods among women, Nigeria, 2017.

The final structural equation model for the full sample indicated that all three constructs of FP social norms were significantly associated with use of a modern contraceptive method (*p* < 0.01) (see Figure 1). For every one-unit increase in FP social norms around delaying an adolescent girl's first pregnancy, there was an increase of 0.11 path coefficients in the respondent using a modern method at the time of the survey (coeff = 0.11; 95% CI = 0.04, 0.19). Note, each FP social norms construct was measured in five units in total, with each increasing unit indicating more favorable social norms (e.g., shifting from agreeing to strongly agreeing that the community approves is an example of increasing social norms, or norms becoming more favorable, by one unit). Modern contraceptive use increased by about 0.31 coefficients for every one unit increase in social norms around using FP to space or limit pregnancies (coeff = 0.31; 95% CI = 0.19, 0.43); and increased by about 0.08 coefficients for every one unit increase in norms around using contraception even when the husband disagrees (coeff =0.08; 95% CI = 0.01, 0.14). Except for household wealth, use of a modern contraceptive was also significantly associated with all covariates (*p* < 0.01) (see Figure 1).

The models differed by location in the FP social norm constructs that were associated with respondents' use of modern contraception (see Table 5). In Kaduna only, the social norm around adolescents delaying their first pregnancy was associated with respondents' use of a modern contraceptive. In Ilorin only, the norm around using contraceptives when a husband disagrees was associated with use of a modern method. In all three sites, the norm around spacing and limiting pregnancies was associated with respondents' use of modern contraception.

**Table 5:** Path coefficients by study location for structural equation model for association of family planning social norms with current use of modern contraceptive methods among women who ever had sex, Nigeria, 2017.

	**Ilorin** ** (*n* = 1,685)**	**Kaduna** ** (*n* = 3,238)**	**Jos** ** (*n* = 1,473)**
**FP social norm factors/constructs for**			
Delaying first pregnancy	0.06	0.25^***^	−0.21
Spacing and limiting pregnancies	0.35^**^	0.24^*^	0.50^***^
Using contraception when husband disagrees	0.14^**^	0.03	0.03
**Covariates**			
Age	−0.15^**^	−0.21^***^	−0.25^***^
Ever married	−0.68^***^	−0.71^***^	−0.29
Highest education	0.06	0.24^***^	0.06
Parity	0.31^***^	0.39^***^	0.40^***^
Religion	−0.19	−0.81^***^	−1.97^***^
Level of religiosity	0.12	0.11	0.68^***^
Household wealth	0.10^*^	−0.01	0.02

* p<0.05;

** p<0.01;

*** p<0.001.

## Discussion and conclusion

After adjusting for demographic characteristics and household wealth among all women who ever had sex in three cities in Nigeria, we found that social norms around using FP to delay first pregnancy, to space and limit pregnancies, and when the husband disagreed with contraceptive use were associated with women's use of modern contraception. However, when we examined the data by level of exposure to the NURHI program (i.e., by city of residence) we found substantial differences. Social norms around using FP to space or limit pregnancies was associated with modern contraceptive use in all three cities. In contrast, FP social norms to delay first pregnancy among adolescents was associated with modern contraceptive use in Kaduna only; and FP social norms for using contraception when a husband disagrees was associated with modern contraceptive use in Ilorin only.

The observed differences suggest that the relationship between specific FP social norms and women's use of modern contraception depends on numerous factors at the individual, interpersonal and societal levels (Rimal and Lapinski, 2015), and this may include malleable factors influenced by the NURHI program. For example, increasing access to FP among youth, including addressing provider bias by using human-centered design approaches was a deliberate focus of Phase 2 and in Kaduna only (NURHI, 2018b). This component of the program may have supported positive FP norms specifically around adolescents' use of contraception, which in turn may have influenced contraceptive use among a broader cross-section of women in Kaduna. This spillover effect is similar to findings from another study in Nigeria using Performance Monitoring and Accountability 2020 data where authors observed a significant relationship between a facility's delivery of adolescent reproductive healthcare and modern contraceptive use by sexually active women of all reproductive ages (Asaolu et al., 2019). Communities are often less accepting of adolescents' use of FP than that of adult women's (Adams et al., 2013; Cannon et al., 2022), and this is supported by our study as well, given that in all three cities the mean scores for social norms supporting adolescents' use of FP was lower than those of the other measured FP social norms. Positive social norms around adolescents using FP to delay a first pregnancy indicates that individuals perceive members of their community to be open to and/or lenient about FP use in general. That is, this specific norm is likely the most “liberal” of perceived FP norms, indicating that women likely believe that their communities would accept most women's use of FP. However, this FP social norm may influence women's actual use of modern contraception only in certain circumstances, such as when supply side factors, such as access to a range of modern methods and quality of services, are improved. While supply side factors were addressed in Ilorin during NURHI Phase 1, they were strengthened in Phase 2 in Kaduna only. A longitudinal study of quality of services in NURHI sites found that in 2017 Kaduna had better quality services and significantly more new contraceptive users compared to Ilorin (Speizer et al., 2019). Improved access and higher quality services may be necessary factors to accompany perceived norms that are favorable to FP in order to influence contraceptive behaviors.

The observed relationship in Ilorin between FP social norms around using contraception when the husband disagrees and use of modern contraception is more challenging to explain. This finding may be a result of socio-cultural differences between the cities. For example, while both Ilorin and Kaduna are a majority Muslim, there are different compositions of ethnic groups between these two States of Nigeria, Kwara and Kaduna States. The majority of residents in Kwara State are Yoruba people while in Kaduna State they are Hausa and Funali. One multi-level analysis of spatial distribution and factors associated with modern contraceptive use among women in Nigeria found that Yoruba women were more likely than Hausa women to use modern contraception (Bolarinwa et al., 2021). Given that our study did not control for ethnicity, this may be a salient factor that influenced our results. Varying gender norms among different ethnic groups may influence whether specific FP social norms are associated with use of modern contraception. Gender norms are informal rules and shared social expectations that distinguish expected behavior based on gender (Marcus et al., 2015) and keep the gender system intact (Cislaghi and Heise, 2019a). Gender norms is one element of the gender system, a social system that apportions resources, roles, power and entitlement according to whether a person or practice is perceived as male or female, masculine or feminine (Ridgeway, 2004). Gender norms are embedded within the institutions and narrative of a given culture, produced and reproduced through individuals' actions, and enforced by those who hold power and benefit from compliance to those norms. As such, gender norms have been predominantly conceptualized as a social construct (West and Zimmerman, 1987; Ridgeway, 2004; Cislaghi and Heise, 2019a). Cislaghi and Heise (Cislaghi and Heise, 2019a) suggests, however, that gender norms are at the intersection of the social and individual because the role they play in shaping women's and men's access to resources affects their voice and sense of self and power.

Social norms around FP—particularly beliefs about the contraceptive behaviors women in a community would or should practice despite disagreement or opposition from their husbands—incorporate gender norms. Perceptions of what community members support and are doing with regard to a gendered behavior depend on societal and cultural expectations of women's and men's roles, how community members occupy those roles, and whether perceptions of community members' occupation of those roles are accurate. Gendered expectations to prove fecundity may dictate that women (and men) refrain from using modern contraception. Gendered expectations to replicate male-dominated power dynamics may dictate that that women use contraception only if their husbands agree. Because social and gender norms and their association with behaviors are contextually specific, it may be possible that a family planning program such as NURHI disrupts unequal gender norms in one city (Ilorin) but not in another city (Kaduna), despite greater intensity of the program.

Some of our findings diverge from previous research results on social norms and contraceptive use. Most salient, our social norm factors for delaying first pregnancy, and spacing and limiting pregnancies include both injunctive and descriptive items. While our original analysis plan was to separate factors by descriptive norms and injunctive norms, doing so did not produce high factor loadings. Though distinguishing between injunctive and descriptive norms has become a common approach to social norms and behavioral research, the practice is not uniformly supported by all behavior change theorists (Rimal and Lapinski, 2015). For example, revisions of the theory of reasoned action do not make a distinction between these types of norms (Yzer, 2012). Relatedly, the fact that retaining injunctive and descriptive items within a single factor produced better statistical results may suggest that some respondents do not conceptually differentiate descriptive norms from injunctive norms.

This study has several limitations. First, this is a cross-sectional study and temporality cannot be established. It is possible that women who use contraception are subsequently more likely to perceive positive norms related to family planning within their communities. Additionally, when asked what most people in their community would do or whether most people in a reference group would approve of a particular behavior (injunctive norms), some respondents may revert to sharing their own opinions and perspectives (Cannon et al., 2022). This means that the survey questions in this study may have captured gender norms [operationalized as attitudes toward gender roles and dynamics in relation to family planning (Cislaghi and Heise, 2019a)] in addition to, or instead of, perceived social norms (perceptions of what community members would do or approve of with regard to family planning).

This study also has several strengths. Compared to running separate regression models, using structural equation modeling is a more comprehensive method to analyzing the relationship among latent variables. Structural equation modeling explicitly assesses measurement errors and estimates latent variables *via* observed variables. Additionally, fully developed models are tested against the data using SEM as a conceptual structure, meaning the relationship among the latent constructs and the observed variables must be pre-specified. The conceptual structure is then evaluated for fit against the sample data (Byrne, 2011).

As part of its FP2030 commitment, the Government of Nigeria aims to increase the national contraceptive prevalence rate from 12% to at least 27% through scaling up evidence-based, high impact practices (FP2030, 2022). The Government has also committed to reducing social and gender norms that hinder access to right-based family planning information and services (FP2030, 2022). Both of these efforts can be supported through continued communications and mobilization interventions that focus on shifting social norms around family planning. As seen in the study results, whether and how social norms shift depend on both the type of norm and the context and population within which the norm is being addressed. In order to design interventions that are adequately nuanced and have the best chance of effectiveness, program designers must differentiate and adapt interventions to specific communities. For example, while promoting norms to use family planning to space pregnancies may be an effective strategy across most communities, promoting norms for family planning use among adolescents may be less palatable and therefore less effective in certain communities. Program designers can use various analytic tools, such as the Social Norms Exploration Tool (Institute for Reproductive Health, 2020), to explore social norms for specific populations and their reference groups, and use the results to help better design norms interventions that are specific for their communities.

## Conclusion

Further research is needed on how collective social and gender norms influence perceived social and gender norms and how both are related to behavior. Challenges remain in measuring collective norms, which exist at a social level, with validity. Aggregating data collected at the individual level is likely to be misleading (Lapinksi, 2005). Additionally, we need a more granular understanding of the circumstances under which positive FP social norms lead to improved reproductive health behaviors. Whether and how norms lead to a given behavior depend on attributes of the behavior, such as how private or detectable the behavior is and how independently of other people the behavior can be carried out (Rimal and Lapinski, 2015; Cislaghi and Heise, 2018); characteristics of the individual, such as self-efficacy and self-monitoring (how strongly people are influenced by personal values and attitudes compared to the behaviors of those around them); and interpersonal- and societal-level moderators, such as group identify and group proximity (Rimal and Lapinski, 2015). The relationship between norms and behavior are further influenced by the strength of sanctions of not following the normative behavior and whether the norm is proximately or distally related to the behavior (Cislaghi and Heise, 2019b). These characteristics must be considered when designing future social and behavioral programming around family planning and reproductive health.

## Data availability statement

The original contributions presented in the study are included in the article/supplementary materials, further inquiries can be directed to the corresponding author/s.

## Ethics statement

The studies involving human participants were reviewed and approved by Institutional Review Boards at the University of North Carolina at Chapel Hill (UNC-CH) (No. 17-1215) in the United States and the National Research Ethics Committee of Nigeria (No. NHREC/01/01/2007). Written informed consent for participation was not required for this study in accordance with the national legislation and the institutional requirements.

## Author contributions

IS and LC contributed to the conception and design of the original study. LC and CM led the data collection. MM led the design and analysis of the secondary data and wrote the manuscript. All authors contributed to manuscript revision and read and approved the submitted version.

## Funding

This work was supported, in whole or in part, by the Bill & Melinda Gates Foundation [OPP1161858]. Under the grant conditions of the Foundation, a Creative Commons Attribution 4.0 Generic License has already been assigned to the Author Accepted Manuscript version that might arise from this submission. The authors also received general support from the Population Research Infrastructure Program through an award to the Carolina Population Center (P2C HD050924) at the University of North Carolina at Chapel Hill.

## Conflict of interest

The authors declare that the research was conducted in the absence of any commercial or financial relationships that could be construed as a potential conflict of interest.

## Publisher's note

All claims expressed in this article are solely those of the authors and do not necessarily represent those of their affiliated organizations, or those of the publisher, the editors and the reviewers. Any product that may be evaluated in this article, or claim that may be made by its manufacturer, is not guaranteed or endorsed by the publisher.

## Author disclaimer

The contents of this article are solely the responsibility of the authors and do not necessarily represent the official views of CPC or the Bill & Melinda Gates Foundation.
